# Culture and gambling fallacies

**DOI:** 10.1186/s40064-015-1290-2

**Published:** 2015-09-17

**Authors:** Li-Jun Ji, Kayla McGeorge, Ye Li, Albert Lee, Zhiyong Zhang

**Affiliations:** Department of Psychology, Queen’s University, 62 Arch St., Kingston, ON K7L3N6 Canada; Department of Psychology, Huazhong Normal University, Wuhan, China; Department of Psychology, Beijing University, Beijing, China

**Keywords:** Gambling, Culture, Gambling fallacy, Hot-hand fallacy

## Abstract

Euro-Canadians and Chinese typically hold different theories about change; Euro-Canadians often engage in linear thinking whereas Chinese often engage in non-linear thinking. The present research investigated the effects of culture-specific theories of change in two related gambling fallacies: the gambler’s fallacy (GF; the belief that one is due for a win after a run of losses) and the hot-hand fallacy (HHF; the belief that one’s winning streak is likely to continue). In Study 1, participants predicted the outcome of a coin toss following a sequence of tosses. Study 2 involved predicting and betting on the outcome of a basketball player’s shot following a sequence of shots. In Study 1, Asians (mainly Chinese) were significantly more likely than Euro-Canadians to believe that they would win (correctly predict the coin toss) after a series of losses (a non-linear thinking pattern), suggesting greater susceptibility to the gambler’s fallacy. In Study 2, Euro-Canadians were more likely than Chinese to predict outcomes consistent with a basketball player’s streaks (a linear thinking pattern), suggesting greater susceptibility to the hot hand fallacy. By illustrating the role of cultural differences in cognition, these findings contribute to our understanding of why certain cultural groups, such as Chinese, are more susceptible to gambling.

## Background

“The only sure thing about luck is that it will change”. – Bret Harte

Imagine yourself standing at a slot machine, pulling the lever and holding your breath as the cartoons spin in front of you, hoping that luck will come through and that you will get three identical pictures in a row. The pictures stop spinning and you realize that you have lost the round, as well as the money you just paid to play. You decide to try again, and you lose once again. Before you know it, you have played ten rounds without any success. Would you keep playing the game after your series of losses? What thoughts or underlying beliefs would motivate your decision to continue or discontinue gambling? Would your justification to continue to play revolve around the idea that your luck is likely to change for the better? Or would you give up gambling because you are feeling like you are having a ‘bad luck day?’ The present research seeks to understand underlying cognitions that may influence patterns of gambling behavior.

Gambling is a common practice in the United States and Canada. Out of 10, 765 American college students surveyed, 42 % had gambled in the past year with 2.6 % gambling weekly or more (LaBrie et al. 2003). The prevalence rate of gambling in Canada in 2006–2007 was 70.7 % (Wood and Williams 2009), with 61.35 % of youth (aged 15–24) indicating that they had gambled in the past year (Huang and Boyer 2007). The high incidence of gambling reflects its status as a common pastime for North Americans and suggests that gambling behavior is an important area of research.

So far, most studies in the gambling literature have examined prevalence and gambling motivation within Western populations. Among ethnic Chinese, the prevalence of problem gambling—defined as a range of gambling issues that influence gamblers’ daily lives, but are not yet at a clinical, or pathological level (Loo et al. 2008)—varies geographically, and although cross-cultural research in gambling prevalence has yielded some inconsistent findings (e.g., Loo et al. 2008), there is evidence that Chinese communities outside of China tend to have greater incidences of problem gambling than the general population. For example, in the United States, Asian students had higher estimated gambling rates (12.5 %) than several other cultural groups (Caucasians, African, Americans, Aboriginal Americans, whose rates were 4–5 %; Lesieur et al. 1991). Similarly, rates of problem gambling were higher in the Chinese-Australian community (Oei et al. 2008, estimated 2.1 %) compared to other cultural communities in Australia (e.g., 1.3 % in Caucasian–Australians; Oei et al. 2008; Victoria Casino and Gaming Authority 2000). A study in Canada produced similar results, with a problem gambling prevalence of 3.0 % in Quebec’s Chinese community compared to the general provincial rate of 2.6 % (CFSGM 1997). Overall, a meta-analysis of 25 studies that examined cross-cultural incidence of gambling behavior found that the problem gambling rate in Chinese communities across the world range from 2.5 to 4.0 % on average (Loo et al. 2008). This trend is consistent with findings that identifying as a minority may increase one’s chance of developing problem gambling behavior (Abbott and Volberg 1994).

The greater incidence of problematic gambling in Asian cultural groups (especially Chinese) generates the following question: What are the cultural variables that are likely to influence gambling behaviors? Much of the cross-cultural gambling literature points to the influence of sociocultural and familial variables. Historical, traditional, and social influences (such as the popularity of dice and card games throughout history in China, or gambling as a central feature in social events and festivals) may be keys to cultural-specific perceptions that gambling is a normal and approvable lifestyle choice (Clark et al. 1990; Victoria Casino and Gaming Authority 2000; Loo et al. 2008). For example, problem gambling behaviors are related to parental gambling (Raylu and Oei 2004; Wynne et al. 1996; Clarke 2004), having a strong Chinese ethnic identity (Lai 2006), acculturative stress (Raylu and Oei 2004; Lee et al. 2007a, b; Jacoby et al. 2013), and limited access to services (Lai 2006). In contrast, there is a dearth of cross-cultural gambling research focusing on the underlying cognitive factors for gambling; the present research seeks to enrich the literature by addressing this gap.

The need to investigate cognitive factors underlying gambling is important because motivational factors are limited in accounting for persistent gambling in light of losses. Although there are external factors (e.g., monetary gains) and internal factors (e.g., amusement seeking; Back et al. 2011; Lee et al. 2007a, b) motivating gambling, non-recreational gamblers continue to gamble even in situations where they consistently lose money. What perpetuates gambling when external motivators, such as monetary gain, are not realized?

To date, there are a number of theories that address this question. Of particular interest in the present study are the gambler’s and the hot hand fallacies. The gambler’s fallacy is the belief that a successful outcome is due after a run of bad luck, or more generally, the belief that a series of independent trials with the same outcome will soon be followed by an opposite outcome [labelled “negative serial dependency” by Goodie and Fortune (2013), Boynton (2003), Burns and Corpus (2004)]. For example, in a fair coin toss, if one gets 4 heads in a row and believes in the gambler’s fallacy, one may expect that a tail is due. This fallacy may explain why addicted gamblers (and lottery ticket consumers) keep gambling despite a series of losses. In contrast to the gambler’s fallacy, the hot hand fallacy is the belief that a successful outcome will follow a run of success (Gilovich et al. 1985; Croson and Sundali 2005; also see Xu and Harvey 2014; Xue et al. 2012). This theory pertains to the belief that a winning streak is likely to continue. For example, if a person has successfully scored several basketball shots in a row, or guessed the outcomes of numerous coin tosses correctly, he or she may be considered “hot” and be expected to continue to succeed in making shots or guessing outcomes. In situations where each trial is independent (e.g., as in Roulette and many other common casino games), the gambler’s and hot hand fallacies lead to misjudging the likelihood of success (Goodie and Fortune 2013). These cognitive distortions may contribute to problem gambling: engaging in the gambler’s and hot hand fallacies have both been strongly associated with problem gambling (Goodie and Fortune 2013).

Research examining cross-cultural differences in gambling fallacies may provide helpful insights into our understanding of gambling behaviors across different cultural or ethnicity groups. Fong et al. (2014) examined Chinese gamblers’ betting decision in a Macau casino, and found that their positive recency bias—believing that the next outcome will be the same as the most recent one (Ayton and Fischer 2004)—decreased as streaks increased. However, they had data from Chinese gamblers only, which does not allow us to draw any cross-cultural conclusions. Empirical support and anecdotal evidence explored by Walker et al. (2006) showed that Chinese gamblers, compared to British gamblers, are more likely to gamble to “test their luck” and tend to put greater weight on their perceived luck over the probability of attaining certain outcomes. Such finding highlights the importance of exploring gambling fallacies in cross-cultural settings.

Another area of research that may provide insight into cultural differences in gambling is culturally specific lay theories of change (i.e., beliefs of how events develop and change over time; Ji et al. 2001; Ji 2005). For example, to Chinese, change typically occurs in a nonlinear—even cyclical—fashion; they tend to believe that what goes up ‘must come down’, or that two people in a bad relationship could become close friends later (Ji et al. 2001). Thus positive events are thought to lead to negative events, and negative events can lead to positive events. In contrast, European North Americans tend to hold a relatively linear theory of change, that is, a belief in either no change, or change only in a linear fashion (e.g., things at rest tend to stay at rest; things in motion tend to stay in motion). Applied to gambling, these lay theories of change suggest that Chinese may be more likely than European North Americans to stop gambling during a winning streak, because Chinese are more likely to hold cyclical theories of change whereas North Americans tend to endorse linear theories of change. However, belief in change may also lead Chinese gamblers to remain hopeful when they lose, perhaps increasing the likelihood of continuous gambling despite a losing streak—this could reflect a manifestation of the gambler’s fallacy.

As cultural beliefs and values may influence gambling behavior at a cognitive level, we seek in the current research to understand more about cross-cultural differences in gambling cognition, in particular the gambler’s fallacy and the hot hand fallacy, by contrasting East Asians (Chinese in particular) and Euro-Canadians. Rather than asking participants outright about their gambling and luck-related beliefs, our method of research examines patterns in gambling behavior from which we extrapolate underlying cognitions. Some researchers have shown that tasks perceived to be random or non-skills based are more likely to generate gambler’s fallacy-consistent expectations (Myrseth et al. 2010), whereas tasks perceived to be less random are more likely to generate hot hand-type expectations (Burns and Corpus 2004). Therefore we designed a seemingly-random task (a coin toss game) to assess the gambler’s fallacy (Study 1) and a scenario involving a game of skill (basketball) to assess the hot hand fallacy (Study 2). Based on differences in lay theories of change, we predicted that Chinese would exhibit a greater tendency than Euro-Canadians to believe that their luck during a coin toss would change, exhibiting the gambler’s fallacy, as assessed in Study 1. We predicted that Euro-Canadians would be more likely than Chinese to believe that a basketball player’s winning or losing streak would continue, exhibiting the hot hand fallacy, as assessed in Study 2.

## Results and discussion

### Study 1: coin toss

Study 1 investigated cultural differences in the gambler’s fallacy using a two-part coin toss game. The game was constructed to investigate how participants would predict future performance in response to patterns of previous wins and losses. We examined whether the gambler’s fallacy could occur after a winning or losing streak and whether there were cultural differences in the gambler’s fallacy.

Because the study design involved both between participant (culture) and within participant (previous outcome and length of streak) variables, as well as a binomial dependent variable (win or loss prediction), a repeated measure generalized estimating equations (GEE) was appropriate for analyzing the data. So, we conducted a 2 (culture: Euro-Canadian vs. Asian) × 2 (previous outcome: win or loss) × 2 (length of streak: 3 or 6) GEE analysis on participants’ predicted outcomes, with culture as the independent variable and previous outcome and length of streak as within participant factors. This analysis allows us to examine the omnibus main effects and interaction effects (see raw frequencies in Table 1).

**Table 1:** Number of participants in Study 1 who predicted a win or loss after a streak of wins or losses (with percentages in brackets)

Prediction	After 3 losses		After 6 losses		After 3 wins		After 6 wins	
	Win	Loss	Win	Loss	Win	Loss	Win	Loss
Caucasian	21 (51.2 %)	20 (48.8 %)	13 (31.7 %)	28 (68.3 %)	26 (63.4 %)	15 (36.6 %)	29 (70.7 %)	12 (29.3 %)
Asian	28 (70 %)	12 (30 %)	21 (52.5 %)	19 (47.5 %)	20 (50 %)	20 (50 %)	24 (60 %)	16 (40 %)

A GEE analysis indicated that there was a significant main effect of previous outcome: participants in general were more likely to believe that they would win after a series of wins than after a series of losses, wald *X*^2^(1) = 14.42, *p* < 0.001, odds ratio = 1.48. This main effect was qualified by a significant two-way interaction between Culture and Previous Outcome, wald *X*^2^(1) = 4.46, *p* = 0.035, odds ratio = 0.73. Specifically, Euro-Canadians believed that they would be more likely to win after a series of wins than after a series of losses, wald *X*^2^(1) = 14.42, *p* < 0.001, odds ratio = 1.48, whereas Asians predicted almost equal chances for a win or loss after streaks of wins, as after streaks of losses, wald *X*^2^(1) = 0.48, *p* = 0.49, odds ratio = 1.08. In other words, Asians were more likely than Euro-Canadians to predict a win after a series of losses, and predict a loss after a series of wins. In addition, there was a marginally significant two-way interaction between Outcome and Length of Streak, wald *X*^2^(1) = 3.36, *p* = 0.067, odds ratio = 0.77, such that longer streaks increased the belief that a trend would continue. No other effect approached significance, wald *X*^2^(1)s < 1.05, *ps* > 0.30.

Participants indicated their confidence on a rating scale from 1 to 7, thus confidence was a continuous (instead of binomial) variable. As a result, a repeated measure ANOVA was appropriate for data analysis. Confidence was analyzed with a 2 (culture) × 2 (previous outcome: wins or losses) × 2 (length of streaks: 3 or 6) repeated measures ANOVA, with culture being a between subjects variable and the latter two variables being within subject variables. The analyses revealed a significant main effect of previous outcome on confidence: participants in general were more confident after a winning streak (estimated mean = 4.52, SE = 0.13) than after a losing streak (estimated mean = 3.97, SE = 0.15), *F* (1, 79) = 19.72, p < 0.001, partial η^2^ = 0.20. They were also more confident after a long streak (estimated mean = 4.37, SE = 0.15) than after a short streak (estimated mean = 4.13, SE = 0.13), *F* (1, 79) = 4.69, p = 0.033, partial η^2^ = 0.06. In addition, there was a trend that Asians (estimated *M* = 4.47, SE = 0.18) were more confident than Canadians (estimated *M* = 4.02, SE = 0.18), *F* (1, 79) = 3.12, *p* = 0.081, partial η^2^ = 0.04. No other effect was significant, Fs < 2.17, ps > 0.14.

### Study 2: basketball

Study 2 was designed to investigate cultural differences between Chinese and Euro-Canadians in the hot hand fallacy, the opposite of the gambler’s fallacy, in a non-random scenario involving skills: we chose to use scenarios involving a basketball player making or missing several hoops in a row.

Participants predicted whether the player would score or miss their next shot following streaks of hits or misses. See raw frequencies in Table 2. Given that participants’ prediction was a binomial variable, as in Study 1, we conducted a 2 (culture) × 2 (previous outcome: hit or miss) × 2 (length of streak: 5 vs. 15) repeated measure GEE on participants’ prediction, with the latter two variables as within participant factors.

**Table 2:** Number of participants in Study 2 who predicted a hit or miss after a streak of hits or misses (with percentages in brackets)

Prediction	After 5 misses		After 15 misses		After 5 hits		After 15 hits	
	Hit	Miss	Hit	Miss	Hit	Miss	Hit	Miss
Caucasian	11 (17.5 %)	52 (82.5 %)	5 (7.9 %)	58 (92.1 %)	54 (85.7 %)	9 (14.3 %)	56 (88.9 %)	7 (11.1 %)
Asian	25 (25 %)	75 (75 %)	21 (21 %)	79 (79 %)	74 (74 %)	26 (26 %)	85 (85 %)	15 (15 %)

The GEE analysis showed a significant main effect of Previous Outcome, such that, in general, more participants predicted hit in response to a hit streak than in response to a miss streak, wald *X*^2^ (1) = 165.518, *p* < 0.001, odds ratio = 2.25. This effect, however, was qualified by an interaction between culture and previous outcome, wald *X*^2^ (1) = 3.75, *p* = 0.053, odds ratio = 0.844. Specifically, Canadians tended to predict a hit after a hit streak and a miss after a miss streak, wald *X*^2^ (1) = 78.18, *p* < 0.001, odds ratio = 1.98, whereas such a tendency was weaker among Chinese, wald *X*^2^ (1) = 55.85, *p* < 0.001, odds ratio = 1.63. The results indicate that Canadians had a stronger tendency than Chinese to predict outcomes consistent with streaks. In other words, Canadians showed a stronger hot hand fallacy for winning streaks (as well as a comparable cold hand fallacy for misses) than did Chinese. No other effects reached significance, wald *X*^2^ (1)’s < 2.21, *ps* > 0.13.

Participants indicated how much money, between 1 and 100 dollars (or Yuan), they were willing to bet on each of their predictions regarding the basketball player’s hits or misses. Bets were treated as an indication of participants’ confidence in their own predictions. Given that different currencies were used in Canada vs. in China, we recognize that direct cross-cultural comparisons on the bets can be problematic. We caution readers that any direct cross-cultural comparisons should be interpreted with prudence. Although the currencies can be converted based on the exchange rate or big mac index, there is no guarantee of their psychological equivalence across cultures. Thus we decided to use the original amount reported by participants as the dependent variable, and examined confidence within each culture separately. Given that bets are measured as continuous variables, we conducted a 2 (previous outcome: hit or miss) × 2 (length of streak: 5 or 15) repeated measures ANOVA within each culture separately. On average, Chinese bet 50.03 yuan (SE = 2.00), and Canadians bet 39.35 dollars (SE = 2.73). Results from two separate repeated measure ANOVAs showed similar patterns of results across the two culture groups. First, both Canadian and Chinese participants bet more money after hitting streaks than after missing streaks. The estimated means for Canadians were 44.37 (SE = 3.01) for the hitting streaks and 34.32 (SE = 3.12) for the missing streaks, F (1, 62) = 12.94, p = 0.001, partial η^2^ = 0.173. The estimated means for Chinese were 55.66 (SE = 2.05) for the hitting streaks and 44.40 (SE = 2.66) for the missing streaks, F (1, 99) = 19.14, p < 0.001, partial η^2^ = 0.162. In addition, participants in general bet more money for a longer streak (15 consecutive hits or misses) than for a shorter streak (5 consecutive hits or misses). The estimated means for Canadians were 47.81 (SE = 3.41) for the long streaks and 30.88 (SE = 2.68) for the short streaks, F (1, 62) = 36.91, p < 0.001, partial η^2^ = 0.373. The estimated means for Chinese were 55.03 (SE = 2.35) for the long streaks and 45.03 (SE = 1.89) for the short streaks, F (1, 99) = 44.92, p < 0.001, partial η^2^ = 0.312. The interaction between previous outcome and length of streak, however, was significant among Chinese, F (1, 99) = 9.23, p = 0.003, partial η^2^ = 0.085, but not among Canadians, F (1, 62) = 0.07, p > 0.78. Specifically, Chinese tendency of betting more after hitting than after missing streaks was stronger for the long streaks, F (1, 99) = 24.25, p < 0.001, partial η^2^ = 0.197, than for the short streaks, F (1, 99) = 6.40, p = 0.013, partial η^2^ = 0.061.

### General discussion

Results from Study 1 showed that Asians were less likely than Euro-Canadians to predict that gambling outcomes would continue linearly. In other words, Asians were more likely than Euro-Canadians to believe that their luck would change during the next round. Asians’ greater tendency to predict a win after a streak of losses indicates that they were more susceptible to the gambler’s fallacy than were Euro-Canadians. In Study 2, likewise, Euro-Canadians were more likely than Chinese to believe that a streak of success or failures would continue in the same vein. This indicates that Canadians were more susceptible to the hot hand fallacy (and its “Cold Hand” Fallacy counterpart for losing scenarios) than were Chinese. Thus we have obtained consistent results in terms of cultural differences in gambling fallacies in both random tasks (coin-toss) and nonrandom tasks involving skills (basketball).

Because losing (or failure) occurs more often than winning (or success) in gambling, this means that Chinese gamblers who are losing may be less likely to believe their losing streak will continue, and may gamble longer and more sums of money than would Euro-Canadian gamblers, which may contribute to the higher prevalence of problem gambling in the Chinese population. The results also support previous research on lay theories of change: Chinese are less likely than Euro-Canadians to make linear predictions (Ji 2005). The findings are consistent with past research (Ji et al. 2008), which has shown that, in the context of stock price prediction and decisions, Chinese demonstrated the gambler’s fallacy beliefs more and the hot hand beliefs less than did Canadians.

Whereas past work has examined situational and individual difference variables influencing gambling fallacies (e.g., Barron and Leider 2010; Roney and Trick 2003), the present research highlights how cultural variables—cultural worldviews of change—may play an important role in gambling fallacies (Raylu and Oei 2004). Such findings have implications for gambling behaviors in general. Future research should further unpack the culture variables and investigate which aspects of culture are responsible for the observed cultural differences.

The present research presents a few limitations. First, the Euro-Canadian sample included mainly women. There is evidence, albeit contradictory, that gender influences rates of problem gambling and endorsement of gambling fallacies. For example, Seutens and Tyran (2012) found that men were more susceptible to the gambler’s fallacy than women, whereas Dohmen et al. (2009) found that men were more likely than women to demonstrate the understanding that a series of chance events are independent. In their systematic review of gambling among Chinese participants, Loo et al. (2008) found that men were at a higher risk of problem gambling than women, yet women experienced worse outcomes of problem gambling. Finally, Fong et al. (2014) did not find an effect of gender on gambling fallacies. The unbalanced gender ratios in the present research, unfortunately, does not allow for a meaningful examination of gender effect. Future studies may focus on how culture and gender interact to influence the endorsement of the gambler’s and the hot hand fallacies.

Second, all participants were university students. Past research suggests that university students in North America have a higher prevalence of problem gambling (Blinn-Pike et al. 2007) and are more likely to endorse the gambler’s fallacy (Marmurek et al. 2014) than members of the general community (e.g., adolescents or adults not in college or university). More research is needed to first investigate whether this phenomenon is present cross-culturally, and secondly, to extend our findings to adults not in university.

Third, we did not include direct measures of cultural values and thinking processes (such as lay theories of change). Future research should include bigger and more balanced samples, and add qualitative measures about cultural beliefs and values so that we can better understand the factors responsible for gambling behavior across cultures. Additionally, it would be useful to complete follow-up studies to better examine behaviors seen in actual gambling environments such as casinos.

The present findings have practical implications for gambling education outreach programs as well as potential treatment avenues. Current treatment programs are not especially equipped to take culture into consideration when designing treatment plans (Raylu and Oei 2002). With this in mind, it may be useful for gambling intervention programs to place an emphasis on educating clients about underlying cultural beliefs influencing gambling fallacies (such as the gambler’s fallacy and the hot hand fallacy), and how these can affect their perceptions of future outcomes. Past research has found that education about mathematical probability in gambling was useful in decreasing the likelihood that participants succumb to gambling fallacies (Williams and Connolly 2006); perhaps education about trends and probabilities of trend reversals may be useful for gambling education for certain treatment groups.

## Conclusions

Overall, the present research has shown that cultural groups experience gambling fallacies differently—Asians are more subject to the gambler’s fallacy and less subject to the hot-hand fallacy than Euro-Canadians, and that these differences are likely influenced by underlying cognitive beliefs that vary across cultures. These findings are an important step in identifying why certain cultural groups, such as Chinese, are more susceptible to gambling. Future research with problem gambling populations is recommended in order to further develop our understanding of the effects of culture and cognition on gambling behaviors.

## Methods

### Study 1: coin toss

#### Participants

We aimed at getting 40–60 participants from each culture group, and recruited 81 participants from a Canadian university, including 41 Euro-Canadian participants (31 women and 10 men) ranging from 17 to 20 years of age, mean = 18, and 40 Asian participants (30 women and 10 men) ranging from 18 to 22 years of age, mean = 19. The majority of them were Chinese or Chinese Canadians (*n* = 37) with 2 Korean Canadians and 1 Japanese Canadian. All participants received course credit for their participation.

#### Materials and procedure

We used Medialab™ version 2010 to program the game. Unsuspecting participants were told that they would be participating in a virtual coin toss guessing game where the coin tosses were randomized by the computer. They were also told that the chances of tossing heads or tails on the computer were the same as they would have been in real life. But in truth, each coin toss sequence was programmed ahead of time to ensure that all participants would encounter the same coin toss sequences.

The game presented participants with a virtual coin and asked them to predict whether it would land on heads or tails. After participants made each prediction, a virtual coin was tossed and the program reported whether they won or lost the toss (i.e., correct versus incorrect in their prediction). There were eight series of coin tosses, each involving three or six guesses each. The computer program predetermined the series of wins or losses, without participants’ knowledge. As a result, in four of the experimental series, each participant got three or six wins or losses in a row, whereas the other four series acted as filler items with a mix of wins and losses to disguise the true nature of the experiment. At the end of each series after learning about the previous outcomes, the computer asked participants whether they thought their next prediction would be correct or incorrect if they were to toss another coin. This prediction served as the main dependent variable. A correct prediction meant a win and an incorrect prediction meant a loss. After making a prediction, they indicated how confident they were on a scale from 1 (not confident at all) to 7 (very confident) about their prediction.

### Study 2: basketball

#### Participants

Participants were recruited from a Canadian university as well as a university from central China. The sample consisted of 63 Euro-Canadian participants (57 women and 6 men) and 100 Chinese participants (52 women and 48 men). Euro-Canadians ranged from 17 to 23 years old, *M* = 18.19 while Chinese participants ranged from 16 to 22 years of age, *M* = 19.12. All participants received course credit for their participation.

#### Materials and procedure

Participants completed a questionnaire about sports gambling, which contained six scenarios describing how many shots a basketball player had made or missed. Among the six scenarios, four were test items and two were filler items. The test items involved a player missing or making consecutive shots (either 5 or 15 times in a row). For example, “A basketball player has made 15 shots in a row without missing in a game (or has missed 15 shots in a row in a game)”. The filler items involved a player’s performance with a mix of hits and misses (e.g., “A basketball player has made 10 shots and 10 misses in the past 20 shots in a game”). The gender of the player in the scenario was not specified. After each scenario, participants were asked to predict if the player would score or miss their next shot. They were also asked to indicate the amount of money, between $1 and $100 (or yuan for Chinese participants), they would bet on their prediction of score or miss. The question implied that the money was their own, but the bets were hypothetical. The monetary bet was used as an indicator of confidence. Lastly, participants answered demographics questions, including gender, age, year of studies and ethnicity. The order of the six basketball scenarios were randomized across participants, resulting in three versions of the questionnaire. (Note: there were no order effects, *p* > 0.129.)

## Abbreviations

ANOVA: analysis of variance
GEE: generalized estimating equations
GF: gambler’s fallacy
HHF: hot-hand fallacy
